# Triple surveillance: a proposal for an integrated strategy to support and accelerate birth defect prevention

**DOI:** 10.1111/nyas.13600

**Published:** 2018-03-13

**Authors:** Lorenzo D. Botto, Pierpaolo Mastroiacovo

**Affiliations:** ^1^ Division of Medical Genetics, Department of Pediatrics University of Utah Salt Lake City Utah; ^2^ International Center on Birth Defects International Clearinghouse for Birth Defects Surveillance and Research Rome Italy

**Keywords:** prevention, neural tube defects, spina bifida, surveillance, folic acid, health outcomes

## Abstract

Preventing neural tube defects (NTDs) easily qualifies as a high‐value opportunity to improve childhood survival and health: the unmet need is significant (major preventable burden), the intervention is transformative (providing sufficient folic acid), and delivery strategies (e.g., fortification) are effective in low‐resource countries. Yet, NTD prevention is lagging. Can public health surveillance help fix this problem? Critics contend that surveillance is largely unnecessary, that limited resources are best spent on interventions, and that surveillance is unrealistic in developing countries. The counterargument is twofold: (1) in the absence of surveillance, interventions will provide fewer benefits and cost more and (2) effective surveillance is likely possible nearly everywhere, with appropriate strategies. As a base strategy, we propose “triple surveillance:” integrating surveillance of cause (folate insufficiency), of disease occurrence (NTD prevalence), and of health outcomes (morbidity, mortality, and disability). For better sustainability and usefulness, it is crucial to refocus and streamline surveillance activities (no recreational data collection), weave surveillance into clinical care (integrate in clinical workflow), and, later, work on including additional risk factors and pediatric outcomes (increase benefits at low marginal cost). By doing so, surveillance becomes not a roadblock but a preferential path to prevention and better care.

## Introduction: the case for surveillance

### Surveillance: not a roadblock but a better path to prevention

In the quest to improve childhood survival and health, preventing neural tube defects (NTDs) easily qualifies as a high‐value opportunity: with regard to preventable burden of disease, the unmet need is significant; a transformative intervention (providing sufficient folic acid) is available; and delivery strategies (e.g., fortification) have proven effective in both high‐ and low‐resource countries.

The unmet need is global, but is particularly high in low‐resource countries,^1^, ^2^, ^3^ where most of the yearly 300,000 NTD‐affected pregnancies occur and where the associated mortality, morbidity, and disability are magnified by the scarcity of surgical and medical care.

**Figure 1:**
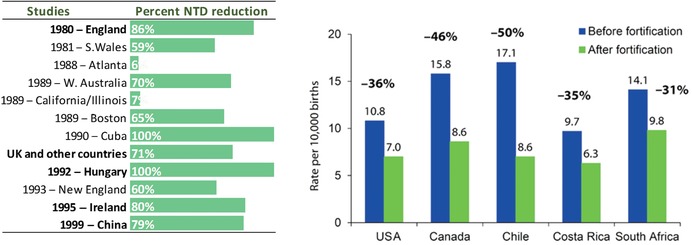
Left panel: percent NTD reduction with oral folic acid supplementation during the periconceptional period. Figure courtesy of Dr. J. Mulinare; redrawn and modified from Ref. 30. Right panel: NTD rate reduction (and percent reduction) after mandatory flour fortification with folic acid. Reprinted from Ref. 31.

This need is tractable. The transformative intervention—providing women of childbearing age with sufficient folic acid, an inexpensive B vitamin—is remarkably straightforward and effective (Fig. 1), and if effectively implemented worldwide could prevent 200,000 cases of NTDs yearly, according to some estimates.^4^, ^5^

Yet, with few remarkable exceptions (Fig. 1), NTD prevention is lagging, spotty, or unsustained,^1^, ^2^, ^4^, ^6^ despite decades‐old evidence that NTD prevention is a tractable challenge with potentially massive benefits worldwide.^4^, ^5^ Many countries have approved regulations to fortify; however, implementation of those regulations is often lagging or incomplete.^7^ Fully realizing the potential for NTD prevention requires a combination of political will, technical capacity, and locally tailored operations management, among other factors, and each of these dimensions includes multiple levels of complexity. The specific question addressed here is how public health surveillance can help support, accelerate, and improve prevention.

### The value of surveillance for prevention

Critics contend that surveillance is largely unnecessary for prevention, that limited resources ought to focus solely on interventions, and that in any case surveillance is too complex to succeed in developing countries. Such criticism should not be disregarded. Indeed, surveillance can become overly complex and will require resources, and, with or without surveillance, prevention interventions will likely have some beneficial effects. Decisions about implementing interventions, such as fortification, may have been driven in different countries by factors other than data on burden of disease. Arguably, science‐based advocacy, together with technical assistance to industry and government, is crucially important in developing, implementing, and regulating large‐scale programs, such as fortification.

However, the view that surveillance is unnecessary or unrealistic misjudges its value as a tool to support and improve interventions. Without a public health surveillance framework, interventions risk being less effective and efficient: they may not live up to their full potential and end up costing more and doing less than they would otherwise. Surveillance can strengthen and focus policy development by increasing awareness of the health burden of NTDs. Following interventions, such as fortification, surveillance helps demonstrate its benefits and detect gaps that would otherwise be missed. Importantly, from a quality improvement standpoint, without a surveillance framework to evaluate outcomes, mistakes are repeated, good intentions flounder, and opportunities for improvement are lost. Surveillance is but a supporting actor in the storyline driven by the protagonists—the actual interventions. But supporting actors make the protagonists shine.

These considerations about challenges and goals lead to three key questions:

1. How can we rethink surveillance so that it can better support and accelerate prevention, including not only primary prevention but also prevention of complications and adverse health outcomes (burden of disease)? Rare conditions, such as specific congenital anomalies, have health impacts that are disproportionately higher than their birth prevalence in terms of morbidity, mortality, and disability. Additionally, not all NTDs can be prevented by folic acid fortification, so improving care is as important a goal for families and the healthcare system as primary prevention.
2. How can a better surveillance framework be applicable not only to NTDs and folic acid fortification but also more broadly to other preventable congenital anomalies and adverse birth outcomes? Systems and data are costly, so maximizing the value of surveillance means leveraging its infrastructure for multiple health purposes.
3. How can such surveillance also be realistic and applicable in low‐resource settings, in a stepwise and practical approach? Systems and goals have to be practical in addition to useful, especially (but not only) in low‐resource settings. Systems developed in developed countries with extensive resources are unrealistic elsewhere. However, limited resources can help spur innovation and focus on what is locally important and possible.

To address these needs, we propose a surveillance strategy based on integrating three components—triple surveillance—while massively streamlining data collection and prioritizing the clinical use of the data. By doing so, surveillance can become not a roadblock but a preferential path to prevention. This approach is novel and has not yet been fully implemented anywhere, though, in a less integrated fashion, some or all of its components have been operational in several countries.

## Triple surveillance: tracking the causal chain

**Figure 2:**
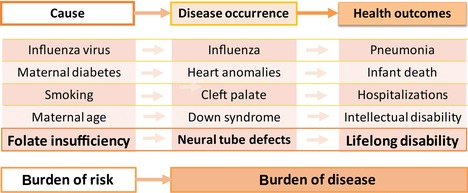
Examples of causal chain, from cause to occurrence to outcomes, for selected congenital conditions.

Public health surveillance has been defined as the ongoing, systematic, and timely assessment of a health condition aimed at generating information that is used for action.^8^ Such action may include interventions to prevent or mitigate adverse health effects, such as immunizations to prevent serious infectious diseases or health diet and exercise to prevent diabetes. This general definition makes it applicable to many public health issues. The key innovation we propose—triple surveillance—broadens the scope of traditional surveillance to include and integrate the three basic domains of the causal chain: from cause to disease occurrence to health outcomes (Fig. 2). One example of a causal chain is smoking causing cleft palate, in turn causing increased childhood morbidity and disability; or smoking causing low birth weight, which causes increased infant mortality; or, in the context of adult disease, smoking causing lung cancer causing increased hospitalizations and premature death. This example, incidentally, also highlights the value of surveillance of risk factors (exposures) shared across several important health outcomes in children as well as adults.

Such integrated triple surveillance can add value to prevention activities in several ways, including the examples summarized in Table 1. One can directly and quickly assess primary prevention interventions by tracking the causal exposures (e.g., smoking) well before such success will translate into better health outcomes (e.g., lower rates of lung cancer). Such quick assessment can also identify problems and gaps (e.g., missed population groups) and accelerate remedies. Additionally, by concurrently tracking disease occurrence and outcomes, interpretations are less prone to errors that can lead to wrong and at times harmful conclusions. For example, decreasing lung cancer rates after implementation of smoking cessation program but without evidence of decreasing smoking rates should prompt an assessment of lung cancer surveillance before blindly announcing success. Finally, tracking health outcomes in addition to disease occurrence (e.g., lung cancer deaths and hospitalizations in addition to lung cancer diagnoses) provides a more precise, realistic, and powerful assessment of the benefits of prevention (or, conversely, the harm of not preventing) that science‐based advocacy groups can leverage to promote further interventions and improvements.

**Table 1:** Benefits and potential challenges of integrating the components of triple surveillance

Surveillance component	Specific benefit	If integrated with other components	Challenge
Blood folate concentration	Measures penetration of intervention in target population: Is sufficient folic acid being effectively delivered to all women of reproductive age?	Helps interpret changes of occurrence rates over time or in specific areas or populations	Sample selection, technical expertise, laboratory capacity, cost, and sustainability (repeated regularly)
Neural tube defect occurrence	Direct assessment of key outcome and major determinant of burden of disease	Provides direct link between treatment and health outcomes (morbidity and mortality)	Population coverage, sustainability, inclusion of stillbirths, and elective termination of pregnancy
Health outcome assessment	Directly assesses the true burden of disease on individuals, families, and society (morbidity, mortality, disability, and quality of life)	Measures true value of prevention, directly usable to improve care	Robust core data set, longitudinal follow up (the longer the better), and complexity

Transposing this approach to NTD prevention illustrates some additional issues—the importance of establishing causality, the usefulness of biomarkers of exposure, and the challenges of disease and outcome assessment—and provides an opportunity to present some operational options to implementation, including in lower resource settings.

### Triple surveillance for neural tube defects

**Figure 3:**
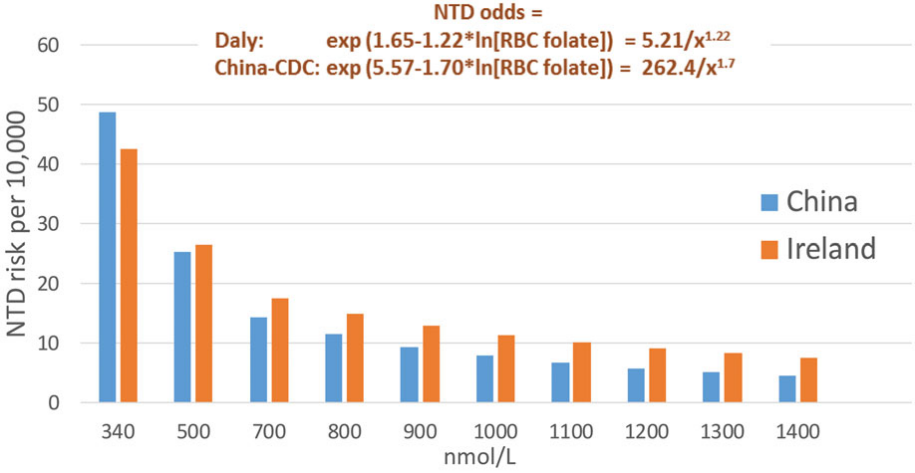
Modeled relation between red blood cell folate concentration (RBC folate) and risk for neural tube defects (NTD). Data are from Refs. 10 and 11.

For folate‐preventable NTDs, the basic question is whether the causal chain (Fig. 2) is established and quantitative. Causality has been long established on the basis of the relation between folic acid use in the periconceptional period and lower NTD risk (Fig. 1). However, the quantitative relation between blood folate concentration and NTD risk is more recent. Accumulating information^9^, ^10^, ^11^, ^12^ indicates that blood (red blood cell (RBC)) folate concentration, rather than only being a general biomarker of exposure (“Are women of reproductive age consuming enough folate?”), is also a quantitative measure of NTD risk (“What is the NTD birth prevalence in a population with a given blood folate concentration?”) and thus an early biomarker of effect. Two studies in particular, from Ireland in the 1990s^11^ and more recently from the China–U.S. Centers for Disease Control (CDC) collaboration,^10^ have generated roughly concordant quantitative models of the relation between RBC folate and NTD risk (Fig. 3). This relationship has major implications for population surveillance of blood folate concentration as the first component of triple surveillance for NTD prevention.

#### Surveillance of blood folate concentration

If blood folate concentration is not only a robust biomarker of exposure but also a predictor of effect, then surveillance of blood folate concentration may be used to estimate population risk (rate) of NTDs (“We have a problem”), even with limited direct NTD data. Blood folate data can also provide an operationally useful preintervention benchmark (“We have a baseline”), and follow‐up blood folate surveys can quickly assess the effectiveness of folic acid interventions (“Is the intervention fully effective, widespread, and equitable?”), months to years before the expected decline in NTD occurrence. This last element is particularly valuable in areas in which surveillance of NTD occurrence is limited or lacking.

#### Surveillance of NTD occurrence

Surveillance of disease (NTD) occurrence, the second component of triple surveillance, is traditionally the more widespread form of surveillance—though even this activity is limited or lacking in most of the world.^2^ In fact, even established systems are often unable to fully assess NTD occurrence metrics, such as birth prevalence (number of cases among a defined cohort of births). Such inability may appear puzzling, as NTDs are clinically obvious and easily and quickly detectable in the newborn nursery or at home without advanced medical training. However, many cases may never make it to birth, either because they result in fetal death (stillbirth) or in elective pregnancy termination after prenatal detection.^13^, ^14^ Thus, tracking NTD occurrence accurately requires including all birth outcomes (live births, stillbirths, and elective terminations of pregnancy for fetal anomalies). Programs that miss stillbirths and pregnancy terminations (and are unable to estimate how many they miss) cannot easily interpret their findings: for example, decreasing NTD rates in live births could reflect the successful outcome of primary prevention due to fortification or supplementation campaigns or, in contrast, more cases detected prenatally and terminated—a very different situation. Also, these data would massively underestimate the burden of disease (especially the occurrence of anencephaly, but also spina bifida), reducing the visibility of NTDs as a health priority and reducing incentives for action. However, even an accurate assessment of birth prevalence provides only a partial and skewed view of the true health burden to people, families, and healthcare systems. A third component, surveillance of outcomes, is necessary to capture morbidity, mortality, disability, or quality of life.

#### Surveillance of outcomes

If surveillance of NTD occurrence is challenging, incomplete, and geographically restricted, surveillance of health outcomes is even more limited, even in high‐resource countries.^1^, ^15^, ^16^, ^17^, ^18^, ^19^, ^20^, ^21^, ^22^, ^23^, ^24^, ^25^ The most commonly reported health outcome is survival, though rarely longer than through infancy or childhood. Broader measures of health, such as morbidity, disability, and quality of life, are seldom assessed systematically, and when they are evaluated it is typically within a specific clinical cohort rather than on a population basis. Moreover, information on outcomes, when available, is often the result of a one‐time study (rather than ongoing surveillance). However, such scarcity is not inevitable: some indicators—whether and when surgery was done, the number and length of admissions, hospital survival—would be readily available in many settings, as they are integral to clinical care and clinical processes. The fact that this information is not readily available or tracked nationally even in high‐resource countries in North America or Europe indicates that scarcity is not due to lack of primary data but due to lack of integration of these data into surveillance.

## Putting triple surveillance into practice

### Massive opportunities for integration and quality improvement

Classical NTD surveillance, which typically focuses on NTD occurrence, provides many benefits where it is deployed. Public health agencies and organizations around the world have promoted, supported, and advocated for public health surveillance locally and internationally. In particular, the U.S.CDC, the International Clearinghouse for Birth Defect Surveillance and Research, EUROCAT, the March of Dimes, and the World Health Assembly have and are playing crucial roles in promoting surveillance as a tool for tackling the challenge of birth defects worldwide.

However, as currently deployed, NTD surveillance has massive margins of improvement. We propose the triple approach strategy as an opportunity to transform traditional surveillance into a practical force to support better primary prevention and improvements in care. To summarize (Table 2), the three components of this approach are (1) surveillance of blood folate concentrations in the population, (2) surveillance of birth defect occurrence, and (3) surveillance of longitudinal health outcomes among those affected. Implementing this system requires true functional integration of the three components (Table 1), particularly in low‐resource settings, and renewed focus on streamlined and efficient data collection and data use (Table 3).

**Table 2:** Neural tube defect (NTD) surveillance throughout the causal chain, at baseline and after interventions

Causal chain	Intervention	→ Cause (marker)	→ Occurrence (prevalence)	→ Outcome (life span)
Surveillance focus		**Blood folate** (in population)	**NTD rate** (all pregnancy outcomes)	**Health, function, and quality of life**
Baseline status	**None** (folate insufficiency)	**↓**	**↑**	**Worse**
After intervention	**Fortification/supplementation**	**↑**	**↓**	**Better**

**Table 3:** Ideal versus current state of surveillance, with potential strategies for improvement

**Surveillance component**	**Ideal state**	**Current state and challenges**	**Strategies/roadmap to start and improve**
Folate sufficiency	Representative, stratified population surveys with ideal methods (RBC folate), tracked over time	Rarely done RBC folate testing complex and costly compared with kit‐based folate measurement Often one‐time surveys, not as part of surveillance	Consider starting with less complex methods (e.g., kit‐based serum survey) for initial tracking (rough estimate) Consider convenience cohorts of women Bundle folate surveys with other nutritional markers for better value and efficiency Incorporate food consumption surveys Expand to other modifiable risk factors of adverse pregnancy outcomes for greater value and efficiency
NTD occurrence	Population‐based ascertainment among all pregnancy outcomes (live births, stillbirths, and pregnancy outcomes), with postnatal follow‐up (e.g., at least 1 year of age)	In many areas, no surveillance and limited health record infrastructure Often only facility based Limited or no capture of home births Often limited to live births Often limited to perinatal period	Start with facility‐based surveillance and live births (if elective terminations of pregnancy are rare or illegal) Consider area sampling to include home births Focus on core key data, usable also for clinical care (no recreational data collection) Integrate surveillance in clinical workflow rather than a separate system, use data also to improve clinical care Expand to other congenital malformations, especially external malformation (low marginal added cost)
Outcomes	Long‐term tracking of multiple outcomes: morbidity, mortality, disability, and quality of life	In many areas, no data Limited or no civil registration (births and deaths) Limited longitudinal health records and system Unstable populations (migration)	Integrate clinics and clinicians into system Use outcome data to assess and improve care Use data for awareness and policy development Integrate data or link to general perinatal and pediatric care systems Expand to include other congenital malformations and key pediatric outcomes (low marginal added cost)

Crosscutting issues:

•Training to build local capacity

•Requires focus and leadership: clear goals, expectations, roles, metrics of success, accountability, and transparency

•Each activity is costly: one must be clear as to “how good is good enough”

### Challenges to implementation

The challenges to implementation must not be underestimated. Some challenges are operational (e.g., cost, organization, and training). Others are related to the ability to observe and report birth defects, such as NTDs. For example, including stillbirths and terminations of pregnancy may require additional sources of information and possibly additional legal authority to collect such information. Some challenges are political: the willingness to invest in preventing birth defects as a sustained health policy.

Further challenges are cultural, because of the novelty of triple surveillance and the emphasis on integrating systems, coordinating activities, and sharing data. Historically, the three components have been and still are the purview of different systems and teams: nutritional epidemiologists design and analyze nutritional surveys, public health epidemiologists focus on birth defect surveillance (often using administrative data), and clinicians in health facilities evaluate health outcomes. Finally, a practical question arises regarding what is feasible and beneficial in a low‐resource country. Even if a single surveillance is absent, how can one even think of establishing triple surveillance?

In discussing a path to effective and sustainable triple prevention, it may help looking at the ideal state and then articulate ways in which that ideal state can be reached, depending on the current state, resources, and priorities in individual countries (Table 3).

### A stepwise approach tailored to local goals and resources

Global success requires not only commitment but also adapting processes so that they are practical, scalable, and effective in a low‐resource setting, where most of the births occur worldwide. Table 3 provides examples of the ideal state of triple surveillance components, with ideas for strategies that can move stepwise from simple but valid operations to more complex and complete processes. Importantly, even simple and less costly assessment, if done properly, can provide significant value for prevention, and in fact can be comparatively rapid and inexpensive. Importantly, implementation in low‐resource settings requires innovative thinking, in particular rethinking what is essential for the overarching goal of improved health outcomes. Table 4 illustrates some broader issues that expand beyond NTD surveillance and prevention and aim at enlarging the scope of triple surveillance to many other risk factors and outcomes. These issues deserve particular attention, especially (but not exclusively) in low‐resource settings because the marginal cost of expanding surveillance to other conditions is comparatively low once an efficient and well‐planned system is in place.

**Table 4:** Overall framework and operational innovations for improved surveillance

Proposal	Why	Comment
A. Overall framework		
Commit for the long term	Good programs take time to work and take root, and surveillance is meaningful if ongoing and sustainable	Commitment also includes training and capacity development and building a surveillance tradition and culture
Integrate surveillance into the clinical workflow	Timeliness and quality can quickly degrade if birth defect surveillance is separate and independent of clinical workflow. However, in many areas, this is precisely what happens (and has happened for decades)	Incorporating key elements of birth defects in existing clinical and public programs (including perinatal surveillance programs) decreases data abstraction and should improve speed and accuracy (timely and accurate data)
Focus on what matters	Choose data for their usefulness—only if meaningful for reporting and ongoing assessment—avoiding “recreational” data collection	Use the system also for perinatal and pediatric surveillance; information fed back to clinicians is crucial for evaluating and improving care and prevention
B. Operational improvements and innovations		
Include all pregnancy outcomes	Include live births, stillbirths, and pregnancy terminations. Stillbirths and terminations are a significant “hidden” toll of birth defects	Stillbirths and terminations of pregnancy are very often undetected, unexamined, and underreported and are often excluded from burden‐of‐disease assessments
Expand surveillance to common adverse outcomes	The system should accommodate clinically important outcomes (e.g., low birth weight), which can be relatively simple to track (e.g., birth weight) and share similar risk factors to birth defects	These outcomes are of clinical and public health interest in many safety studies, including of maternal medications and immunizations. Specific tools derived from statistical process control can help monitor several of these additional outcomes
Expand surveillance to important exposures and risk factors	Tracking exposures in the underlying birth cohort provides a comparison group (“controls”) for risk factor assessment and functions as an ongoing risk factor surveillance system (e.g., for smoking, diabetes, and immunizations)	Tracking modifiable risk factors is relevant for many birth defects, adverse pregnancy outcomes (e.g., low birth weight), and related pediatric health, thus expanding the usefulness of the system. Examples of such risk factor surveillance include the World Health Organization's STEPS and the CDC's PRAMS
Develop a practical and robust sampling frame for large populations	Population‐based surveillance for an entire large LMIC (e.g., India) is extremely challenging, and the data may not be reliable. A sample of small areas rigorously chosen to be representative of the larger region and population(s) can provide reliable, high‐quality data at a fraction of the cost	Sampling of population‐based area surveillance has been used in other contexts (e.g., immunizations and nutrition) to obtain good‐quality information with fewer limitations than convenience‐based facility (hospital) programs, in particular in areas with a large proportion of home births

So, how can we implement triple surveillance in the field? Tailoring operations to the situation on the ground is crucial. Adapting operations and aligning program goals with the community can speed its deployment, lower its cost, and improve data quality. Important practical considerations include the proportion of home births, the availability and use of prenatal diagnosis and pregnancy terminations, the presence and quality of existing surveillance programs and ongoing nutrition and risk factor surveys, and the availability of health records and vital registration systems. In addition, strategies, such as geographic sampling (not discussed further here), can improve efficiencies by focusing limited resources to selected areas; if appropriately done, sampling can help generate high‐quality information—complete, accurate, and timely—that can be used to describe the country at large. When planning the components of triple surveillance, some general strategies can prove invaluable.

### Use what is available

In practice, where some form of surveillance is already in operation, it should be leveraged. Simple systems often have one or more of these characteristics: a hospital‐based network, usually in the form of a convenient sample of all area hospitals; inclusion of hospital births only, with home births missed; limited detection of stillbirths, with those not occurring in the hospital missed; limited follow up, typically from delivery to hospital discharge; and a single source of ascertainment: the maternity hospital itself. While acknowledging the limitations, these existing systems provide helpful data and can be a powerful stepping stone for an expanded and improved program.

### Simple can be fast and good enough

One must not undervalue the importance of initial, rapid assessments and simple approaches (Table 3). Simple blood folate surveys using commercial kits for serum folate can provide rapid and comparatively inexpensive baseline data on folate sufficiency in the population. Likewise, basic nutritional surveys and facility‐based record review for NTD occurrence, morbidity, and mortality can start to build the data framework of a particular area or country, from exposure to outcomes. In some countries, the effect of fortification on mortality and hospitalizations was evaluated using such data.^26^

### Build on success and improve stepwise

Moving forward, one may envision a tiered approach (Table 3). For example, it is entirely reasonable to start with surveillance of birth prevalence of a few major external malformations (or even NTDs alone) and then develop and integrate key elements of triple surveillance (key exposure and key health outcomes). Tools and manuals to develop and improve surveillance of birth defect occurrence are available.^27^, ^28^, ^29^

For example, one might link birth defect surveillance to locally organized or national surveys on risk factors. In several low‐ and middle‐income countries, representative nutritional and risk factor surveys are carried out periodically, at times including biomarkers (e.g., of nutrition). This component, integrated with the NTD occurrence and outcome data, is essential for triple surveillance.

To improve the assessment of disease occurrence, one might expand the network to more hospitals, ideally a thoughtful sample of hospitals in representative areas, until all the geographic area is included, including home births.^27^, ^28^, ^29^ This step should help decrease referral bias and improve the representativeness of the findings. Selected elements to improve surveillance of NTD occurrence are listed in Table S1 (online only). In a large country, one could also consider using sampling as a way to explicitly and systematically select representative geographic areas in which to focus resources for surveillance: by doing so, it may be possible to obtain high‐quality and representative information with comparatively limited resources.

To better evaluate outcomes, one might incorporate hospitals beyond maternity units, in particular those that care for affected infants (e.g., pediatric hospitals), and link with vital records data. Also, to evaluate and improve outcomes, the system could be linked to services within the healthcare system. Because treatment services in lower resource areas are scarce and patchy, surveillance should add value to the clinicians, facilitating the referral to care. Early referral to the appropriate medical and surgical services is a major determinant of survival and morbidity. Developing partnerships between NTD surveillance programs and services, such as surgery, can in turn enhance the surveillance of outcomes.

## Conclusions: not done but doable

Most of the world's pregnancies with NTDs occur in areas with limited or no surveillance. By focusing on what matters—cause, NTD occurrence, health impact—the framework of triple surveillance can help countries efficiently plan, deploy, and assess interventions and lower the cost of prevention. It is worth underscoring that, although each component will provide benefits, the real synergies occur when they are integrated.

These strategies require leadership and commitment, but the payoff can be considerable. This framework, importantly, has two additional benefits. First, it can (and should) be expanded to other exposures, conditions, and outcomes. The marginal cost of expansion would be comparatively small, at least initially, as many risk factors are relevant to multiple conditions that affect both pediatric and maternal health (e.g., maternal diabetes, smoking, and nutrition), and adding other external malformations to NTDs in surveillance systems is relatively straightforward. Second, a closer integration of exposures, disease occurrence, and health outcomes supports an explicit shared focus on prevention and improved care. A renewed emphasis on including tangible markers of outcomes will help improve treatment and clinical care, a key driver of population health.

Finally, it is fair to ask an additional key question. Should prevention strategies, in the form of mandatory fortification or supplementation, await the full deployment of the triple approach? Our answer is an emphatic no. One would not delay care to set up the perfect hospital surveillance infrastructure. Likewise, prevention is so beneficial, in terms of lives saved and population health, that delays are ethically unacceptable. However, not trying to improve strategies is also fundamentally problematic, as it perpetuates waste and negates benefits to some (or many). Development of a surveillance infrastructure could be reasonably begun concurrently with interventions. For example, folic acid fortification, though conceptually simple, in practice takes time: policies need to be approved, funded, organized, and deployed. In the case of mandatory fortification, a reasonable time (typically years) is given to millers to comply with regulations. Such unavoidable lag can (should) be used to complete a simple blood folate survey among women of reproductive age and assemble a core surveillance system for NTD occurrence and outcomes.

Ultimately, the larger issue is rethinking surveillance to increase the effectiveness and efficiency of prevention and care. We believe that the strategy of triple surveillance is both valuable and doable, provided it is focused, streamlined, accepted, and tiered. Besides its practical early benefits, triple surveillance supports a link that often goes missing in public health surveillance: the integration of public health and clinical activities and goals. By supporting such integration, surveillance can become not a roadblock but a preferential path to prevention and better care.

## Competing interests

The authors declare no competing interests.

## Supporting information


**Table S1**. Selected considerations to promote high‐quality surveillance of occurrence of neural tube defects (NTDs) and by extension of many other major congenital anomalies.
