# Supplementary material for: Triple surveillance: a proposal for an integrated strategy to support and accelerate birth defect prevention
Source: Ann N Y Acad Sci. 2018 Mar 13;1414(1):126–36. doi: 10.1111/nyas.13600 (PMC5873412; doi:10.1111/nyas.13600)
Supplement: Supplementary file 1 — Table S1. Selected considerations to promote high‐quality surveillance of occurrence of neural tube defects (NTDs) and by extension of many other major congenital anomalies. [file NYAS-1414-126-s001.docx]

**Triple Surveillance –Supplementary Table**

Supplementary Table 1. Selected considerations to promote high quality surveillance of occurrence of neural tube defects (NTDs) and by extension of many other major congenital anomalies.

| **Action** | **Comment and examples** |
| --- | --- |
| Include livebirths, stillbirths, pregnancy terminations | - Including only livebirths results in underestimates and potentially in significant bias (in the presence of pregnancy terminations) - Stillbirths can include many cases of anencephaly especially - Inspection of stillbirths for NTDs and other malformation can be difficult (depending on context and culture) but very important - Facility based surveillance likely misses many stillbirths - Pregnancy terminations, where these occur, can account for many cases of NTDs; including pregnancy terminations is a priority for areas with high rates of prenatal diagnosis and legal abortion |
| Include hospital and home births | - Starting with hospital (facility)-based reporting is typical in many low and middle income countries (LMIC): these can provide important information, but the larger the proportion of home births, the more difficult it is to interpret the findings in a hospital based system - A system may start as a hospital-based network, with the goal of eventually including first all hospitals in an area, then including also home births in the area - The advantage of hospital based system is typically the availability of hospital (medical records) |
| Include all resident births in an area | - This is the definition of population-based system; it is often thought of as the gold standard for surveillance, with fewer opportunities for bias (e.g., bias associated with referral to specific hospitals) and more likely to generate representative findings (by its ability to capture events in the entire resident population - Population-based systems are the goal of surveillance. Area sampling can be a tool to efficiently generate generalizable information for the population. - Population-based systems are not necessarily good. Good surveillance also requires a focus on all other elements of quality, including complete detection of cases, complete and accurate data collection, and so on. |
| Use of multiple sources of ascertainment | - Multiple sources improve completeness and accuracy - Note that hospital based systems are typically single source systems, in which the source is the maternity hospital/ward - With multiple sources of ascertainment, it becomes crucial (and at times difficult) to ensure that cases are not duplicated. In turn this requires appropriate identification of cases |
| Use standard, reproducible case definitions (inclusions, exclusions) | - Case definitions must be explicit, clear and operational, with inclusions and exclusions - Training and period retraining are crucial: training can be done in person or through online systems |
| Describe phenotype accurately and completely | - Often the person collecting information is not the same as the person coding and classifying the phenotype: accurate and complete description are crucial, including (for NTDs) the location, presence of skin covering, and associated anomalies (related or unrelated to the primary NTD): standards and recommendations are available (see WHO/CDC/ICBDSR surveillance manual for details) - Photographs can be extremely useful, especially for later review on the part of specialists (e.g., clinical geneticists) |
| Code and classify cases appropriately | - Coding and classification of NTDs is relatively straightforward but there are pitfalls that can affect significantly the findings (e.g., the inappropriate management of cases of spina bifida occulta). - Relevant to evaluation of health impact is the appropriate clinical classification of NTD-affected babies, including the presence of clinical sequences (e.g., hydrocephalus, pes equinovarus supinatus), and presence of associated anomalies and syndromes (more common in encephalocele, but also occurring with spina bifida occasionally) |
| Link to vital records | - Vital records are crucially important to systematically and accurately describe denominators and vital status (at delivery and later) - A major challenge in many low income countries is the lack of vital records (medical as well as civil birth and death registration): this challenge is being addressed through several interventions but remains an ongoing concern |
